# Metabolic carbonyl reduction of anthracyclines — role in cardiotoxicity and cancer resistance. Reducing enzymes as putative targets for novel cardioprotective and chemosensitizing agents

**DOI:** 10.1007/s10637-017-0443-2

**Published:** 2017-03-10

**Authors:** Kamil Piska, Paulina Koczurkiewicz, Adam Bucki, Katarzyna Wójcik-Pszczoła, Marcin Kołaczkowski, Elżbieta Pękala

**Affiliations:** 1 Department of Pharmaceutical Biochemistry, Faculty of Pharmacy, Jagiellonian University Medical College, Medyczna 9, 30-688 Kraków, Poland; 2 Department of Medicinal Chemistry, Faculty of Pharmacy, Jagiellonian University Medical College, Medyczna 9, 30-688 Kraków, Poland

**Keywords:** Anthracyclines, Cardiotoxicity, Resistance, Pharmacokinetics, Drug metabolism, Anticancer agents

## Abstract

Anthracycline antibiotics (ANT), such as doxorubicin or daunorubicin, are a class of anticancer drugs that are widely used in oncology. Although highly effective in cancer therapy, their usefulness is greatly limited by their cardiotoxicity. Possible mechanisms of ANT cardiotoxicity include their conversion to secondary alcohol metabolites (i.e. doxorubicinol, daunorubicinol) catalyzed by carbonyl reductases (CBR) and aldo-keto reductases (AKR). These metabolites are suspected to be more cardiotoxic than their parent compounds. Moreover, overexpression of ANT-reducing enzymes (CBR and AKR) are found in many ANT-resistant cancers. The secondary metabolites show decreased cytotoxic properties and are more susceptible to ABC-mediated efflux than their parent compounds; thus, metabolite formation is considered one of the mechanisms of cancer resistance. Inhibitors of CBR and AKR were found to reduce the cardiotoxicity of ANT and the resistance of cancer cells, and therefore are being investigated as prospective cardioprotective and chemosensitizing drug candidates. In this review, the significance of a two-electron reduction of ANT, including daunorubicin, epirubicin, idarubicin, valrubicin, amrubicin, aclarubicin, and especially doxorubicin, is described with respect to toxicity and efficacy of therapy. Additionally, CBR and AKR inhibitors, including monoHER, curcumin, (−)-epigallocatechin gallate, resveratrol, berberine or pixantrone, and their modulating effect on the activity of ANT is characterized and discussed as potential mechanism of action for novel therapeutics in cancer treatment.

## Introduction

Anthracyclines (ANT) are a class of cell-cycle non-specific anticancer antibiotics that were first isolated from the *Streptomyces* genus in the early 1960s. This highly efficacious group of drugs have been commonly used in oncology for over 40 years. The classic ANT, doxorubicin (DOX) and daunorubicin (DAUN), were the first ones employed in cancer treatment and are still frequently used as both monotherapies or in chemotherapy regimens [1]. Several other ANT have also been developed as potent anticancer agents, such as epirubicin, idarubicin, valrubicin, amrubicin, and aclarubicin. Moreover, there is great interest in the development of novel ANT as effective chemotherapeutics. However, this group of drugs is not without flaws. The characteristic and dose-limiting factor of ANT treatment is its cardiotoxic effect. It is estimated that in DOX therapy used at approved doses, the acute form of cardiotoxicity affects ~11% of patients, while the chronic form affects ~1.7% of patients. ANT-induced cardiotoxicity is manifested by arrhythmias, myocarditis, dilated cardiomyopathy, and congestive heart failure [2]. Many potential mechanisms of this adverse effect have been postulated, but the etiology remains unclear. Most reports have focused on theories associated with the generation of reactive oxygen species and the disruption of intracellular ferric homeostasis.

Other studies, however, have postulated that the formation of ANT metabolites — products of a two-electron reduction — secondary alcohols, which are reported to be more cardiotoxic than their parent compounds, are responsible for these adverse effects [3, 4]. Their generation is catalyzed by cytosolic enzymes — carbonyl reductases (CBR) and aldo-keto reductases (AKR). Furthermore, metabolic reduction of ANT has been identified as an important process underlying the resistance of cancer cells [5]. As such, CBR and AKR inhibitors are hypothesized to have cardioprotective and chemosensitizing properties [6, 7].

To-date, no review article has focused specifically on the significance of reductive metabolic pathways of ANT in cardiotoxicity and the development of resistance in cancer cells. The aim of this paper is to provide a comprehensive summary of literature relevant to this topic. The data presented in this article is focused primarily on the most studied ANT, DOX. However, the importance of reductive metabolism in for other ANT is also reviewed. Lastly, the cardioprotective and chemosensitizing activities of reducing enzyme inhibitors and their potential as drugs is discussed.

## Doxorubicinol formation and pharmacokinetics

**Fig. 1:**
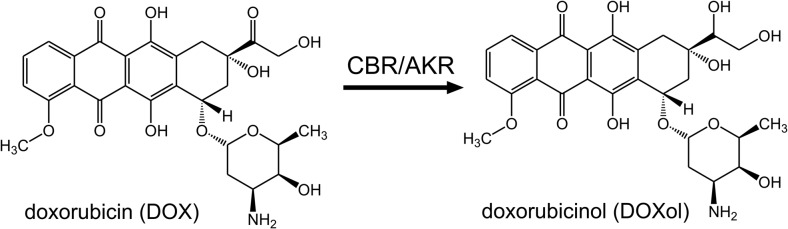
Two-electron reduction of DOX

The main product of a two-electron DOX reduction is doxorubicinol (DOXol) (Fig. 1). The potential role of this metabolite in cardiotoxicity was first proposed in the mid-1980s [3, 4]. While other metabolites are generated at low levels, DOXol is the main metabolite of DOX. The plasma level of DOXol in relation to DOX is inconstant and characterized by large inter-individual variability. In a study involving 18 patients, the average DOXol/DOX AUC (area under the curve) ratio was 0.514 [8].

The liver is the dominant organ responsible for DOXol formation, followed by the kidneys [9]. The results of studies concerning the distribution of DOXol in tissues, especially in cardiac tissue, are inconclusive. Some studies have found accumulation of DOXol in the heart [3, 4], while others have not [10]. A post-mortem study in patients who had received DOX before death observed that a significantly higher concentration of DOX and DOXol was found in the myocardium than in other tissues. The concentration of DOXol in the heart was comparable to the concentration of DOX [11].

DOX is a well-established substrate of ABC transporters, and many studies have indicated DOXol is also suitable substrate for ABC-mediated efflux. In clinical trials, co-administration of the multi-drug resistance (MDR) modulators, such as zosuquidar, with DOX, led to increased exposure to DOX and DOXol [12]. When applying the classic MDR modulator, cyclosporine, the AUC of DOX and DOXol increased by 55 and 350%, respectively. Likewise, an exacerbation of treatment toxicity was observed [13].

Preclinical studies have suggested ABC-mediated transport is important in DOXol distribution. The ABCB1 (P-glycoprotein) inhibitor, nilotinib, was found to alter DOX and DOXol distribution in tumor-bearing mice. In particular, it increased DOXol accumulation in heart tissue, and intensified necrosis and vacuolization in cardiomyocytes [14]. In mice lacking *ABCB1*, prolonged DOX and DOXol exposure in cardiac tissue, as well as in the brain and the liver, was observed [15]. Moreover, studies conducted on cells transfected with *ABCB1*, have indicated DOXol as a better substrate than DOX [16].

Co-administration of MDR modulators with DOX had been found to lead to meaningful changes in DOXol pharmacokinetics, especially in its distribution. Increased accumulation of the metabolite in cardiac tissue, may lead to increased cardiotoxicity of therapy. However, these observations require clinical confirmation in humans.

DOXol is produced by a two-electron, NADPH-dependent reduction of the DOX side-chain carbonyl group (C13) to a secondary alcohol. Two enzymatic pathways for this process have been postulated, i.e. a reduction catalyzed by carbonyl reductases and/or by members of aldo-keto reductase family [17].

### Carbonyl reductases

Carbonyl reductases (CBR) are monomeric, cytosolic enzymes that belong to the family of short-chain dehydrogenases/reductases. CBR catalyze NADPH-dependent reduction of carbonyls to related secondary alcohols. Four isoenzymes have been distinguished: CBR1, CBR2, CBR3 and CBR4. Numerous studies have found CBR1 and CBR3 contribute to the formation of DOXol [9, 17]. Recently, CBR4 was proposed to have ANT metabolizing properties [18]. Kinetic studies have found higher intrinsic clearance of DOX by CBR1 than by CBR3 [9]. The significance of CBR1 in DOX reduction has been demonstrated in animal models. In mice with one functional copy of the *CBR1* gene, the DOXol/DOX ratio was ~60% lower as compared to the control group and the cardiotoxic effects of DOX were also reduced. Moreover, *Cbr1* +/− mice were better protected against DOX-induced mortality and weight loss than the wild type [19]. While mice with overexpression of human *CBR* tended to develop cardiotoxicity more often than the control group [20].

Single nucleotide polymorphisms (SNP) in *CBR* have been investigated in several research studies involving human subjects and were found to influence the pharmacokinetics and toxicity of the enzyme. A SNP in *CBR1* increased DOX-related risk of cardiomyopathy [21], while other SNPs were associated with significantly increased DOX exposures in a population of Asian cancer patients [22]. Additonally, a SNP in *CBR3* increased the risk of cardiomyopathy [21]. The above-mentioned studies demonstrate that CBR activity influences DOX pharmacokinetics, metabolism and toxicity. However, to further confirm this hypothesis, further studies, especially clinical trials with larger samples sizes, are required.

### Aldo-keto reductases

Aldo-keto reductases (AKR) are a superfamily of NAD(P)H-dependent oxidoreductases. Similarly to CBR, these enzymes are monomers which occur in the cytosol and have the ability to reduce ketones and aldehydes to their corresponding alcohols. AKR1C3 (17β-hydroxysteroid dehydrogenase type V) is considered as the main DOX reductase. Other AKR enzymes characterized as DOX reductases mainly belong to the AKR1 (AKR1A1, AKR1B1, AKR1C2, AKR1B10) and the AKR7 (AKR7A2) families [9, 18]. Phenobarbital, an AKR inhibitor, has been shown to influence DOX pharmacokinetics and demonstrate significant cardioprotective properties in rodents [23].

As with CBR, several SNPs in AKR genes which alter their enzymatic activity have been identified. These several *AKR1C3* variants showed a significantly weaker ability to reduce DOX [18], and in patients with such a phenotype, treatment with DOX was correlated with an increase in objective response rate (not statistically significant), progression-free survival, overall survival, and hematological toxicity of chemotherapy [24].

### Which enzyme is a target for modulation of DOX activity?

The enzyme most essential in the process of DOX reduction is not yet determined. However, this is important for the development of novel inhibitors of DOXol formation. Based on the catalytic efficiency of recombinant enzymes, AKR1C3 has been indicated as the most effective reductase in two in vitro studies. Other enzymes with distinguished efficiencies are CBR1, CBR3, CBR4 and AKR7A2 [18, 21]. However, expression levels are also an important factor which influences the significance of enzymes in vivo. The protein level of CBR1 or AKR7A2 in the heart exceeded the protein level of AKR1C3 [18]. While level of enzymes in tumors is inconstant and may be closely related to the type of neoplasm and its exposure to therapy. Based on the studies to-date, determination of the most essential enzyme is not possible.

## Cardiotoxic activity of DOX and DOXol

Although many mechanisms have been postulated, the etiology of ANT cardiotoxicity remains unexplained. The most common hypotheses point to the pro-oxidative iron dependent and independent properties of ANT. However, for the secondary alcohol metabolite of DOX, a somewhat different influence on cardiac tissue has been observed. In isolated rabbit heart muscle, DOXol depressed systolic myocardial function 30 times more potently than DOX. Moreover, a variable influence on ion pumps in cardiac muscle has been observed. DOXol, in contrast to DOX, was a potent inhibitor of Ca^2+^, Mg^2+^, and Na^+^/K^+^-ATPases [4, 25]. In another study, DOXol, at 100 times lower concentration than DOX, was found to produce a similar contractile depression of a rabbit atria as DOX. While in the same concentration, it increased calcium release from sarcoplasmic reticulum vesicles 3–15 times more potently than DOX [26]. Hanna et al. found a comparable influence of DOX and DOXol on RyR2 (ryanodine receptor 2) activity, however, only DOXol affected the SERCA2A (sarco/endoplasmic reticulum Ca^2+^-ATPase) function. These proteins, acting as a receptor and an ion pump, respectively, play a crucial role in regulating the exchange of calcium ions between cytoplasm and sarcoplasmic reticulum [27]. These effects may explain the disruption of cytosolic calcium ions level induced by DOXol. The calcium level is crucial factor in the function of cardiomyocytes. Calcium ions are the link between electric stimulation and cell contraction. Moreover, they modulate the activity of several proteins, contribute in the initiation of apoptosis, and play a role in the pathogenesis of heart diseases [28]. Therefore, disruption of the main calcium regulatory systems may be considered as one of the most important mechanisms of DOXol cardiotoxicity.

Interestingly, the redox theory was also found to be related to the formation of reduced ANT metabolites. DOXol disunites iron ions (Fe^2+^) from iron-sulfur clusters [4Fe-4S] of cytoplasmic aconitase, with its simultaneous reoxidation to DOX. In contrast to physiological conditions, iron-lacking apoprotein-aconitase was unable to activate pathways of iron homeostasis and its iron-reincorporating properties were impaired [29]. These results suggest DOXol may also participate in the hypothesized mechanisms regarding pro-oxidative and iron-related mechanisms of DOX cardiotoxicity.

## Significance of DOXol formation in cancer resistance

Resistance to chemotherapeutic drugs has been recognized as a major factor influencing the failure of cancer therapy and is an obstacle in successful treatment of numerous types of neoplasms. Cancer cell resistance is a significant problem among patients treated with ANT [12, 13, 30]. A major mechanism found to play a dominant role in this phenomenon is the active transport of drugs across the cell membrane. This involves members of the ABC protein family (ATP-binding cassette) i.e. proteins that occur in cell membranes. Their role in resistance is based on the efflux of small-molecular compounds out of the intracellular space. Inhibitors of ABC pumps, such as ABCB1, have been proposed as compounds which may reverse resistance. ABC transporter inhibitors have shown high efficacy in in vitro and in vivo models, however, in clinical studies, they were not sufficiently effective to increase the response to treatment or exhibited adverse reactions [30]. Due to the lack of effective methods which target drug resistance using ABC transporter modulators, research has focused on other drug targets.

The CBR and AKR enzymes, due to their presumed role in ANT detoxification in cancer cells, are potential targets for novel drugs. The secondary metabolites produced by these enzymes have been found to be less cytotoxic than their parent compounds. For example, the LC_50_ values of DOXol were 59–162 times higher than for DOX [5]. Interestingly, in a study by Heiben et al., DOXol exhibited a one-million times lower cytotoxicity than DOX in a clonogenic assay [31].

It has been observed that cancer cells exhibit increased levels of CBR and AKR expression, and these may influence DOX activity. The relative abundance of CBR and AKR enzymes in nine different cell lines were correlated with DOX cytotoxicity and the rate of DOX reduction. Rapidly metabolizing cells, which had higher levels of AKR and CBR, were more resistant to DOX than slowly metabolizing cells [5]. Overexpression of DOX-metabolizing enzymes was found in both primary cancer cells and cell lines. AKR1C3 overexpression was shown in explants taken from patients with breast cancer and in the breast cancer cell line MCF-7 with developed resistance to DOX [32]. Microarray analysis was used to compare gene expression between DOX-resistant and non-resistant MCF-7 cells. The most over-represented genes in resistant cells were members of the ABC family, as well as *AKR1B10* and *AKR1B1* [31]. Furthermore, studies on CBR/AKR inhibitors have confirmed the chemosensitizing properties of such compounds.

**Fig. 2:**
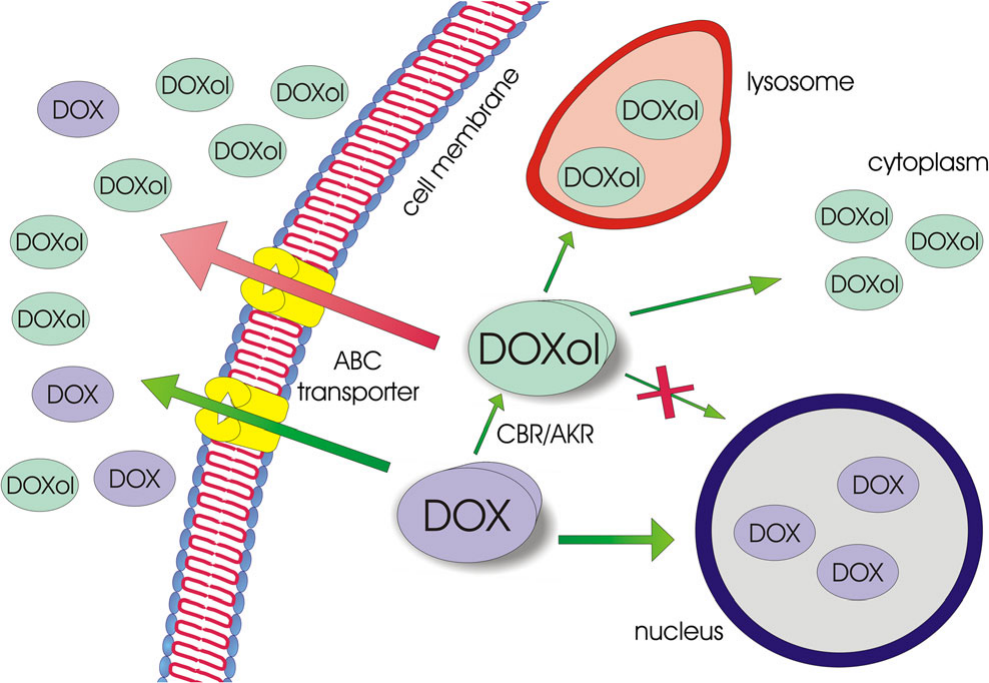
Decreased activity of DOXol may result from increased efflux, intracellular distribution outside the nucleus, and decreased affinity to DNA

As previously described, DOXol seems to be more suitable for ABC-mediated efflux than DOX. A cell line transfected with the *ABCB1* gene, demonstrated 18.9 times higher resistance to DOXol as compared to the parental line. Similar results were observed with DOX, however, the acquired resistance was lower. Treatment of transfected cells with an ABC modulator resulted in increased intracellular accumulation of both DOX and DOXol [16]. In vivo, in tumor-bearing mice, nilotinib, acting as ABCB1 inhibitor, was found to increase the accumulation of DOX and DOXol in cancer tissues [14]. This implies that weaker anticancer activity of DOXol may be related to its increased affinity to ABC transporters, thus leading to a lower intracellular concentration of the agent. On the other hand, DOXol has been shown to have significantly lower DNA binding activity as compared to DOX. Moreover, while DOXol is retained in the cytoplasm or lysosomes, DOX is mainly accumulated in the nucleus [31]. Therefore, it seems that lower DOXol activity may result from both changes in molecule interaction with transporters and molecular targets of DOX (Fig. 2).

## Other ANT — are their alcohol metabolites important?

The literature is much poorer in regards to information on alcohol metabolites of ANT other than DOX. Although knowledge one these drugs are not comprehensive, general conclusions can be drawn.

### Daunorubicin

Carbonyl reduction is the main pathway of DAUN metabolism. The role of daunorubicinol (DAUNol) formation in the activity of DAUN has been investigated in several in vitro and in vivo studies involving cell lines and rodents.

Unlike DAUN, the concentration of DAUNol in cardiac tissue and plasma of rabbits treated with DAUN was correlated with decreased papillary muscle contractility. Furthermore, in contrast to DAUN, the metabolite inhibited calcium uptake into the cardiac sarcoplasmic reticulum [33]. The influence of DAUNol on the cytosolic calcium level has been demonstrated, and it likely occurs through binding of the ANT with calsequestrin — a calcium binding protein [34].

Studies have indicated DAUNol formation may also play a role in cancer resistance. After transfection with the *CBR1* gene, decreased cytotoxicity of DAUN was observed in K562 leukemia cells. In these cells, 94% of DAUN was converted to DAUNol after 2 h of incubation [35]. A valuable study by Varatharajan et al. was conducted on primary acute myeloid leukemia (AML) cells derived from human patients. The study demonstrated a correlation between *CBR1* and *CBR3* expression, DAUNol intracellular level, and decreased DAUN cytotoxicity [36]. Studies have shown a correlation between cytotoxicity and DAUN metabolism in cancer cell lines. An increased reduction rate results in a decreased response of cancer cells to DAUN [5].

### Epirubicin

Epirubicin (EPI) differs from DOX only in the conformation of a carbon atom in a sugar, daunosamine. Pharmacological significance of EPI reduction is only briefly mentioned in the literature. The metabolite has been reported to be less cytotoxic than EPI [37], suggesting a possible role of reduction in resistance development. However, this topic needs further investigation.

### Idarubicin

Idarubicinol (IDAol) is the main metabolite of idarubicin (IDA) in humans. IDA was found to be reduced to IDAol in a perfused rat heart, and the CBR and AKR inhibitors rutin and phenobarbital, respectively, decreased IDAol formation [38]. Weiss et al. in rats observed cardiotoxic properties of IDAol, via an increased coronary vascular resistance than that of IDA. IDAol was also more active in reducing atrial contraction in the perfused rat heart [39].

Little is known about the role of CBR and AKR in IDA reduction. AKR1C3 was found to be IDA reductase and was responsible for IDA-resistance development [40]. However, cytotoxicity studies with the isolated metabolite indicated only a small decrease in activity as compared to the parent compound, incomparably smaller than DOXol and DAUNol [16, 41].

On the other hand, active efflux of IDAol from leukemia cells could be inhibited by MDR modulators, while IDA efflux under the same conditions was not significantly affected [42]. Also, in K562 cells, resistance acquired by *ABCB1* transfection affected IDAol activity more than IDA activity [41]. It may be concluded that IDA reduction may play an important role in resistance, mainly by facilitating drug transport out of cells.

### Valrubicin

Valrubicin (VAL), N-trifluoroacetyladriamycin-14-valerate, acts as a prodrug. Hydrolysis of the ester bound to active N-trifluoroacetyladriamycin and valerate allows N-trifluoroacetyladriamycin to be reduced to N-trifluoroacetyladriamycinol [43]. The pathways involved in this process are unknown. The reduced metabolite has potential anticancer activity, however, based on the existing studies, it is not possible to determine the significance of N-trifluoroacetyladriamycinol in the overall pharmacological effect. VAL is used in the treatment of bladder cancer, and thus it is administered intravesically, with minimal absorption to systemic circulation.

### Amrubicin

Amrubicin (AMR) is an ANT used primarily in Japan for the treatment of lung cancer. Like other ANT, its metabolism has been found to influence its activity. Surprisingly, the toxicity induced by the metabolite was associated with hematological disturbances rather than with cardiotoxic effects [44].

The anticancer activity of the AMR alcohol metabolite is different from other ANT. A significant increase of cytotoxicity after carbonyl reduction has been observed. Amrubicinol (AMRol) was 5–54 times more potent than AMR against several cancer cell lines [45]. An in vivo study with tumor-bearing xenografts revealed a dependence between AMR efficacy and intratumoral metabolism of the drug to AMRol [46].

Similar to IDA, AMR was resistant against P-glycoprotein-mediated efflux to a greater extent than AMRol. Cell transfection with the *ABCB1* gene influenced AMRol cytotoxicity more than the parent molecule [47]. No detailed kinetic studies with recombinant enzymes, which could indicate the dominant enzymes involved in AMRol formation, have been conducted.

### Aclarubicin

In a review of the literature, no metabolite of aclarubicin (ACLA) reduced in its aglycone part has been described to-date. This fact may result from a shift in the carbonyl group from the side chain to the neighboring additional side chain (methyl carboxylate moiety). ACLA metabolism is based on modification and cleavages in its sugar chains, and yields active glycosides and inactive aglycones [48].

## CBR and AKR inhibitors as modulators of ANT activity

Based on CBR- and AKR-mediated production of secondary alcohol metabolites of ANT, the inhibitors of these enzymes have been evaluated as potential cardioprotective agents, with simultaneous chemosensitizing activity. The compounds characterized as CBR/AKR inhibitors include well known agents, already widely studied in pharmacological research. Some of these agents have been tested in clinical trials.

**Fig. 3:**
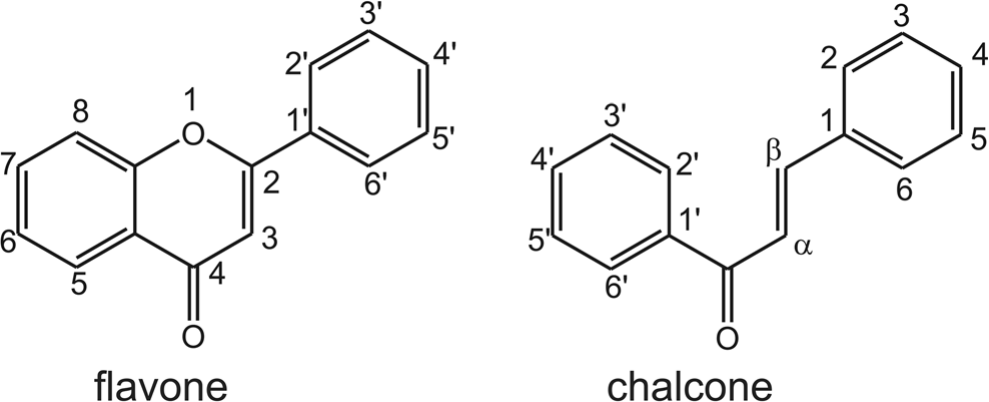
Flavone and chalcone structures

Flavonoids, a class of secondary plant and fungus metabolites with a broad spectrum of biological properties, have been described in many studies as CBR and AKR inhibitors [49–51] and have synergistic properties with ANT. In a Structure-Activity Relationship (SAR) analysis involving 27 flavonoids, assisted by molecular docking, Arai et al. found that the group of flavones (2-phenyl-1-benzopyran-4-one) exhibited the strongest inhibitory properties against CBR1 (Fig. 3). These flavones bind to the CBR1 enzyme through the hydroxyl groups at C5 and C7, and through the carbonyl group at C4, however, the 7-hydroxyl group was critical for potent inhibition. Consequently, the most active compound was luteolin [49].

The properties of the flavonoid CBR1 inhibitor, monoHER, a semi-synthetic monohydroxyethyl derivative of rutoside, were evaluated in a clinical trial in combination with DOX. Administration of monoHER during DOX therapy increased the response to treatment in patients with metastatic soft-tissue sarcoma. However, the number of participants in this study was very limited [52].

Chalcones (Fig. 3) are nature-derived compounds with a wide range of biological activities and evident anticancer properties. A study assessing SARs by Silvestrin et al., based on the inhibitory properties of 12 chalcone derivatives, indicated the requirement of the hydroxyl group at C4′ to inhibit the formation of a secondary alcohol. From hydroxyl-substituted chalcones at C2, C3, C4, C2′ and C4′, the most active compound was 2′,4′,2-trihydroxychalcone. This compound demonstrated non-competitive inhibitory properties in heart cytosolic fraction with an IC_50_ = 21.2 μM (quercetin IC_50_ = 13.7 μM) against DOX reduction [53].

(−)-epigallocatechin gallate (EGCG), a catechin derived from tea (*Camleia sinensis*), has been described as a CBR1 inhibitor. It decreased the reduction rate of DAUN and induced a 16.2-20.5% enhancement of drug cytotoxicity in hepatoma cell lines. This effect did not occur in Hep3B cells with lower CBR1 expression levels. A co-treatment of xenografts (mice) with EGCG and DAUN, resulted in a decrease of tumor volume and reduction of cardiotoxicity. EGCG analogues were assayed, with derivatives without a gallate moiety found to be much weaker inhibitors [7].

Resveratrol, a stilbenoid from grapes, was characterized as a mixed-type inhibitor of CBR1 with a K_i_ = 55.8 μM. Resveratrol and a series of its analogues (4′-metoxy; 5-metoxy; 4′-amino) showed comparative inhibitory properties toward CBR1. However, a 3,5-dimetoxy derivate and resorcinol, a moiety of resveratrol, had no inhibitory activity. The authors concluded that the m-hydroquinone moiety is required for inhibitory properties of the molecule, of which one of the hydroxyl groups is involved in binding with the enzyme [54].

Curcumin (diferuloylmethane) has been employed in cancer therapies in human subjects and is a lead structure for many compounds with anticancer properties. It showed inhibitory properties against CBR1 with K_i_ = 233 nM with a non-competitive inhibition type [55]. Another nature-derived compound, emodin, showed inhibitory activity toward CBR1 (K_i_ = 0.219 μM), AKR1B10 (K_i_ = 0.667 μM) and AKR1C3 (K_i_ = 5.46 μM), significantly increased cytotoxicity of DAUN against A549 and HepG2 cell lines, and inhibited DAUNol formation in lysates obtained from these cells [56].

A pentacyclic triterpenoid, 23-hydroxybetulinic acid, after intragastric administration in mice, reduced intrinsic clearance of DOX from the cytosol of liver and heart cells. It also decreased DOXol concentration in tissues and exerted cardioprotective effects [5]. Moreover, studies using a combination of 23-hydroxybetulinic acid with DOX found synergistic cytotoxic and antitumor properties [57].

Berberine, an isoquinoline alkaloid, prevented DOX-induced decrease of body weight and increase of mortality in male Sprague–Dawley rats. It also decreased the level cardiac injury biomarkers and ameliorated cardiac dysfunction in echocardiographic examination. Decreased DOXol formation and accumulation in the heart were observed during co-treatment with berberine [58]. Among the 19 other isoquinoline alkaloids, stylopine and canadine showed inhibitory properties toward AKR1C3-mediated reduction of DAUN [59]. Oracin, also an isoquinoline derivative developed as an anticancer agent, inhibited DOX reduction in the cytosol of MCF-7 cells and increased DOX cytotoxicity. Moreover, in combination with DOX, oracin demonstrated proapoptotic properties [60].

**Fig. 4:**
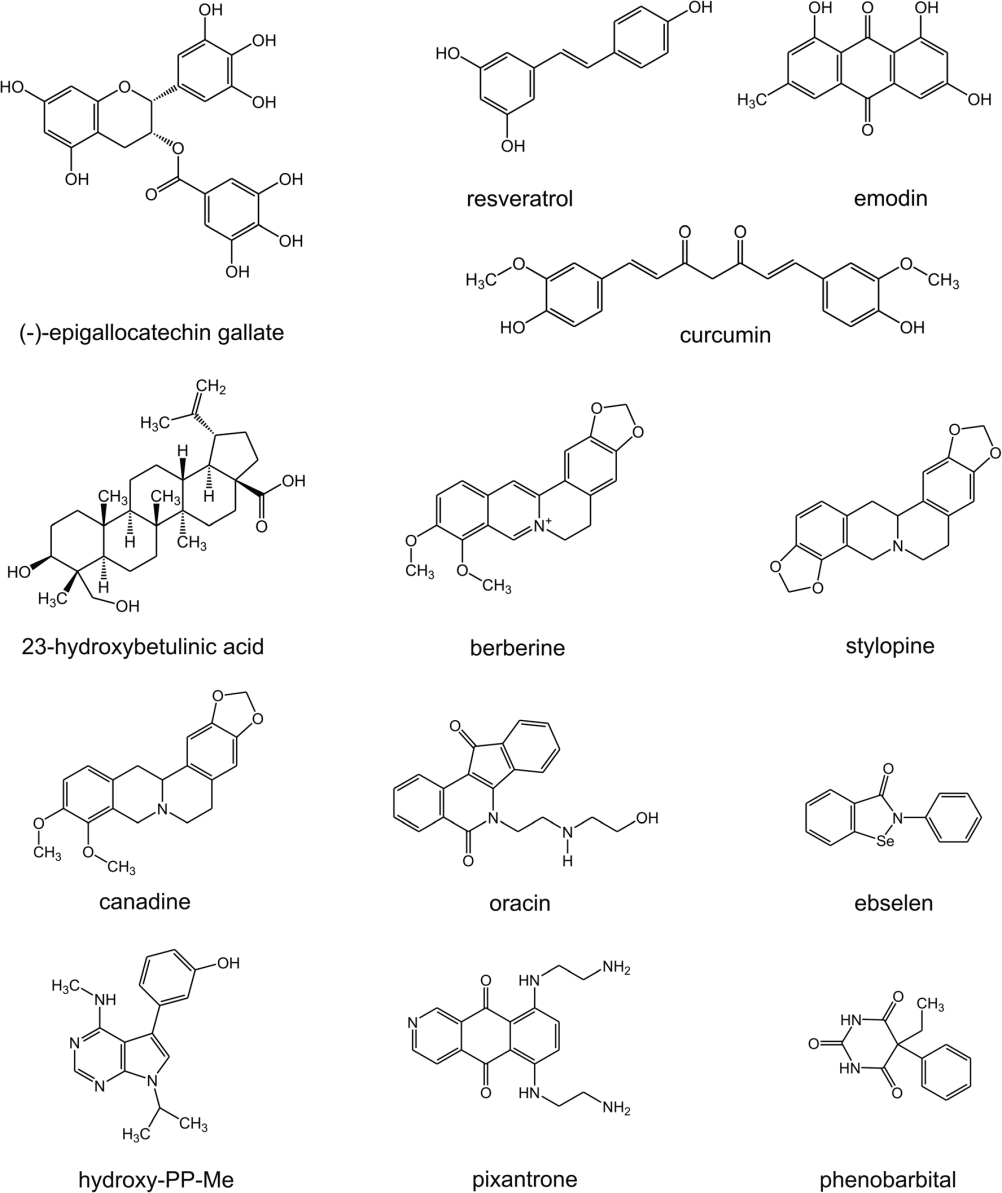
Molecular structures of various CBR and/or AKR inhibitors

Inhibitors of carbonyl reducing enzymes were also found among synthetic structures. An organoselenium drug, ebselen, a cytoprotective, anti-inflammatory and antioxidant agent, has been found to be a non-competitive inhibitor of DOXol formation in the cytosol [61]. Hydroxy-PP-Me, a derivative of the Src kinase inhibitor PP2, through CBR1 inhibition, induced a 25% increase of DAUN cytotoxicity in A549 cells [62]. Inhibitory properties toward reductases were also found for pixantrone. Pixantrone and its metabolites, especially N-dealkylated, showed competitive inhibition of DOX reduction [63]. Phenobarbital, an anxiolytic, anticonvulsant, and sedative drug, influenced DOX reduction in vitro, in heart and liver cell cytosols; in DOX-treated male Sprague Dawley rats, it increased AUC and T_0,5_ of DOX and decreased the serum creatine kinase (CK) level by approximately 50% [22]. The structures of the above described compounds are presented in Fig. 4.

Significant progress in the field of structural biology has led to the identification of several crystal structures representing reducing enzymes. Among them, several high-resolution structures of CBR1 have been solved. The enzymes were co-crystallized either solely with NADP^+^/NADPH cofactor (PDB ID: 3BHI) [64], or together with glutathione (crystals 3BHJ and 4Z3D) [64, 65] and its S-hydroxymethyl derivative (3BHM) [64] and product of formaldehyde adduction to glutathione, BiGF2 (2PFG) [66]. Crystal structures of CBR1 were often elucidated together with an inhibitor hydroxy-PP complexed in the glutathione binding site placed next to the NADP^+^ (1WMA [149] and the complexes: 3BHJ and 3BHM). The experimental structures deposited in the RCSB PDB database were frequently used in molecular modeling studies. The exact crystal structures enabled the development of various conformational models of the enzyme, which in turn allowed for defining the common binding mode of many of the inhibitors.

**Fig. 5:**
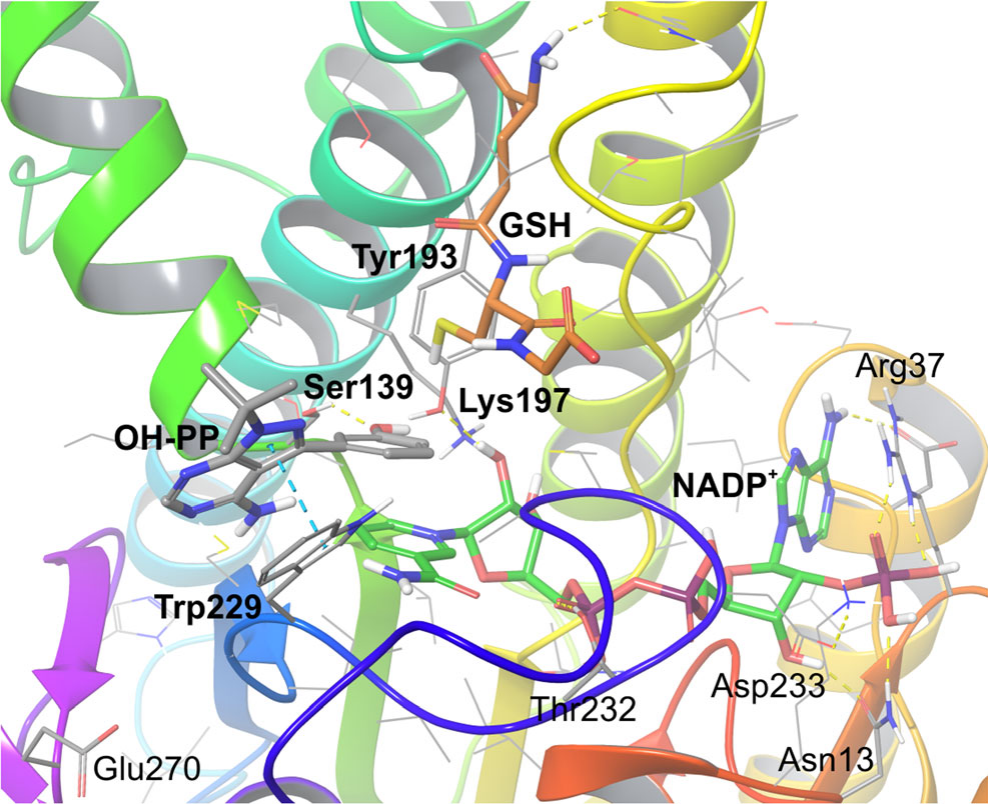
Crystal structure of CBR1 protein (3BHJ) [64]. NADP^+^ cofactor bound in the active site of the enzyme. Substrate mimic inhibitor OH-PP bound in the glutathione (GSH) binding site near the catalytic triad amino acids: Ser139, Tyr193 and Lys197. Amino acid residues engaged in ligand binding (within 4 Å from the ligand atoms) are displayed as sticks, whereas crucial residues, i.e. forming hydrogen bonds (dotted yellow lines) and π-π stacking (dotted blue lines), are represented as thick sticks

The molecular requirements for CBR1 inhibitors and the structural determinants of the enzyme’s active site might be presented in the example of the 3BHJ crystal structure. It represents a functionally complete enzyme, which is complexed with NADP^+^ cofactor and adjacent glutathione, which in turn forms adducts with substrates under physiological conditions. The crystal was obtained with hydroxy-PP, found between the glutathione binding site and the NADP^+^, where residues forming the catalytic triad are exposed. The three crucial amino acids are as follows: Tyr193, which transfers electrons from NADPH to the substrate; Ser139, which stabilizes the carbonyl moiety substrate; and Lys197, which forms hydrogen bonds with the nicotinamide ribose moiety (Fig. 5) [62]. Competitive inhibitors interact mainly with Tyr193 and Ser139, forming hydrogen bonds, as well as additional aromatic interactions (*π*-*π* stacking) with Trp229 [7, 49, 51]. However, 23-hydroxybetulinic acid was found to interact with Trp229 and Glu270 only [6]. On the other hand, a non-competitive inhibitor, curcumin, occupied the binding site of NADPH [55]. Due to the potentially high compliance of computational models based on crystal structures with experimental data, molecular modeling might be regarded as a useful tool for efficiently aiding in the design of novel enzymatic inhibitors.

## Conclusions

Reports of decreased cardiotoxicity of ANT in rodents with *CBR1* gene knockout [19], in rodents co-treated with CBR and AKR inhibitors [6], and in patients with lower activity of enzymes [21] provide pharmacological evidence for the role of selected secondary alcohol metabolites in the development of the cardiotoxic effects of ANT. Reducing enzymes generating secondary alcohol metabolites are overexpressed in resistant cancer cells, leading to an increased rate of ANT reduction [5]. Most secondary metabolites have shown lower cytotoxic activity and increased susceptibility to efflux through ABC transporters. However, there is very limited data concerning the significance of reducing enzymes in the resistance observed during clinical treatment. Multiple studies, including in vivo models, indicate promising ANT activity-modulating properties of CBR and AKR inhibitors. Therefore, a strategy based on inhibition of reductive metabolism could be considered as an approach leading to both a decrease in cardiotoxicity and a decrease in cancer cells resistance.

In summary, ANT reducing enzyme inhibitors should be considered as novel resistance reversing and cardioprotective agents. As other proposed drugs targeting various mechanisms of resistance and cardiotoxicity have thus far not been approved for clinical use, further studies should investigative the clinical efficacy of these reducing enzyme inhibitors.
